# Intranasal dantrolene nanoparticles inhibit lipopolysaccharide-induced depression and anxiety behavior in mice

**DOI:** 10.1038/s41398-026-03816-x

**Published:** 2026-02-03

**Authors:** Jia Liu, Yan Lu, Piplu Bhuiyan, Jacob Gruttner, Lauren St. Louis, Yutong Yi, Ge Liang, Huafeng Wei

**Affiliations:** 1 Department of Anesthesiology and Critical Care, Perelman School of Medicine, University of Pennsylvania, Philadelphia, PA 19104 USA; 2 Department of Anesthesiology, The Affiliated Hospital of Qingdao University, Qingdao, Shandong 26600 P. R. China; 3 Department of Anesthesiology, Shanghai Key Laboratory of Maternal Fetal Medicine, Shanghai Institute of Maternal-Fetal Medicine and Gynecologic Oncology, Shanghai First Maternity and Infant Hospital, School of Medicine, Tongji University, Shanghai, 200092 China

**Keywords:** Depression, Psychiatric disorders

## Abstract

This study investigates the therapeutic effectiveness of intranasal dantrolene nanoparticle pretreatment to inhibit lipopolysaccharide (LPS)-induced pathological inflammation, synapse destruction, and depressive and anxiety behavior in mice. B6SJLF1/J adult mice were pretreated with intranasal dantrolene nanoparticles (dantrolene: 5 mg/kg), daily, Monday to Friday, 5 days per week, for 4 weeks. Afterwards, mice were treated with a single intraperitoneal injection of LPS (5 mg/kg). Behavioral tests for depression and anxiety were performed 24 h after the one-time LPS injection. Biomarkers for pyroptosis-related inflammation cytokine levels (IL-1β and IL-18) in the blood and brain were measured using enzyme-linked immunosorbent assay (ELISA) and immunoblotting, respectively. Changes in primary protein (NLRP3: NLR family pyrin domain containing 3, Caspase-1, N-GSDMD: N-terminal protein gasdermin D) and synapse protein-related (PSD-95 and synaptin-1) activation of inflammatory pyroptosis in mouse brains were measured using immunoblotting. Results indicated that intranasal dantrolene nanoparticle treatment robustly inhibited LPS-induced increases in depressive and anxiety behavior, LPS-induced pathological elevation of IL-1β and IL-18 levels in the blood and brain, and LPS-induced activation of pyroptosis. Furthermore, intranasal dantrolene nanoparticles significantly inhibited decreased PSD-95 and synaptin-1 levels. Intranasal dantrolene nanoparticles have demonstrated neuroprotective effects against inflammation-mediated depression and anxiety behaviors and should be studied further as a future effective drug treatment of major depressive or anxiety disorders.

## Introduction

Three hundred million people worldwide suffer from major depressive disorder (MDD) [1]. MDD is a chronic and recurrent disease affecting about 20% of the population and is the global leading cause of suicide [2]. MDD imposes a tremendous psychological burden, as well as significant social repercussions and contributions to other disabilities [3]. The expected direct and indirect costs of MDD are up to $6 trillion in the US alone [4]. An estimated 4% of the global population currently experiences anxiety disorders. In 2019, 301 million individuals reported suffering from an anxiety disorder, with a lifetime prevalence of approximately 34% [5]. MDD and anxiety disorders were twice as prevalent among women [6, 7], with overall age-specific rates relatively stable or increasing across the lifespan [8]. Depression and anxiety are the most common psychiatric diseases, chronic diseases with unclear mechanisms of pathology [9], and co-exist frequently.

Current antidepressant treatments suffer from significant limitations, including slow onset of action, high rates of nonresponse, and acute worsening of anxiety [10]. Benzodiazepines are ineffective in some anxiety spectrum disorders and are not recommended for long-term use due to concerns about their potential for abuse, tolerance, and withdrawal [11]. Treatment of MDD faces a high risk of resistance (up to 30% of patients are unresponsive to first treatment) and relapse (up to 8%) [3, 9]. Treatment-resistant anxiety with remission rates may be as low as 25–35%, and relapse rates post-remission may reach 30% within a decade [12]. Thus, there is an urgent need to develop novel approaches to treat depression and anxiety, especially treatment-resistant depression or anxiety, with minimal side effects or organ toxicity.

Ketamine, an N-methyl-d-aspartate (NMDA) glutamate receptor antagonist, was approved in 1970 by the Food and Drug Administration (FDA) for use in children and adults as a general anesthetic. Recent studies indicate that ketamine is effective in treating MDD, especially treatment-resistant depression (TRD) [13, 14]. Ketamine is also effective in treating anxiety disorders, even those resistant to traditional treatments [15, 16]. While traditional antidepressants can take weeks to months to have an effect, ketamine has a rapid impact on mood and suicidality, with mood changes reported as early as 4 h after treatment [17]. As an N-methyl-D-aspartate receptor (NMDAR) antagonist, a primary glutamate receptor on the plasma membrane that causes Ca^2+^ influx into the cytosol from the extracellular space, ketamine is a safe and broadly effective drug for treating patients with depression or anxiety disorders, similar to esketamine [18]. This indicates that glutamate-mediated excitotoxicity and its associated disruption of intracellular Ca^2+^ homeostasis play a key role in the pathology of depression and anxiety psychiatric disorders.

Increasing evidence suggests that upstream disruption of intracellular Ca^2+^ homeostasis, together with the associated downstream inflammation and synapse dysfunction, plays a critical role in MDD pathologies [19–21] and anxiety disorders [22, 23]. Overactivation of the ryanodine receptors (RyR) in MDD and excessive Ca^2+^ release from the endoplasmic reticulum (ER) results in depletion of ER Ca^2+^ and pathological elevation of cytosol and mitochondrial Ca^2+^ concentrations, which is detrimental to synapse function and cell survival [21, 24, 25]. RyR overactivation was previously demonstrated to increase anxious behavior as well [24]. Upstream Ca^2+^ dysregulation results in mitochondrial dysfunction [26], mitochondrial and cellular oxidative stress [9, 27], the activation of inflammasomes, and cell and neuron death via pyroptosis [28–31]. This results in the release of inflammatory cytokines (IL-1β and IL-18) and pathological inflammation-mediated cell death [30, 32]. Pathological cytokines, especially pyroptosis-related IL-18, play important roles on inflammation mediated neurodegeneration in psychiatric disorders [33, 34]. Gut dysbiosis and associated inflammation further contribute to observed pathologies in depression and anxiety behaviors [35, 36]. Thus, a drug that inhibits upstream Ca^2+^ dysregulation, thereby limiting downstream pathological inflammation, programmed cell death by pyroptosis, and synapse destruction, is expected to effectively treat MDD and anxiety disorders.

Dantrolene, a RyR antagonist, is an FDA-approved drug for the treatment of malignant hyperthermia, muscle spasms, and neuroleptic syndrome, with tolerable side effects and occasional liver toxicity at high doses [37]. Dantrolene, given its ability to inhibit upstream critical Ca^2+^ dysregulation, is neuroprotective against many neurodegenerative diseases, including cerebral ischemia [38, 39], Huntington’s disease [40], spinocerebellar ataxia [41], amyotrophic lateral sclerosis [42], and seizures [43, 44]. Dantrolene robustly inhibited up to 78% of NMDA-induced elevation of cytosolic Ca^2+^ concentration in cerebral cortical neurons [45], suggesting its high potential to treat MDD, operating with a similar molecular mechanism as ketamine ketamine [46]. These findings suggest an urgent need for further investigation.

In this study, we investigated the therapeutic effectiveness and potential mechanisms of intranasal dantrolene nanoparticles on depression and anxiety behaviors associated with pathological inflammation in adult mice. We induced inflammation via a one-time intraperitoneal injection of LPS. Our results demonstrated that intranasal dantrolene nanoparticle treatment for twelve consecutive weeks robustly inhibited LPS-induced depression and anxiety behaviors in adult mice. These effects were attributed to its robust inhibition of LPS-induced elevation of pyroptosis-related inflammation cytokines IL-1β and IL-18 in the blood and brain, as well as the loss of synapse proteins (PSD-95 and synpatin-1) in the brain. This study indicates that intranasal dantrolene nanoparticles may be an effective treatment for depression and anxiety psychiatric disorders through inhibiting pathological inflammation and synapse destruction in the CNS.

## Materials and methods

### Animals

All procedures were approved by the Institutional Animal Care and Use Committee (IACUC) at the University of Pennsylvania. B6SJLF1/J adult mice were obtained from Jackson Lab. Mice were divided into different cages according to age and gender. There were no more than five mice per cage. Both male and female mice were used in this study. Some mice were excluded based on abnormal appearance, behavior, or body condition following established criteria [47] or as deemed unhealthy by animal facility veterinarian.

### Experimental treatment groups

As demonstrated in Supplemental Fig. 1, adult male and female mice (5-10 months old) were randomly divided into experimental groups. Littermates were used within each experimental group. Non-littermates with identical genetic background, age, and breeding environment were used among different experimental groups without randomization. The mice were pretreated with either intranasal dantrolene nanoparticles (IN Dan, 5 mg/kg) or the empty vehicle (Veh), once a day, 5x/week (Monday through Friday), for four continuous weeks. The purpose of this pretreatment was to optimize therapeutic efficacy and assess potential side effects, given the lack of prior studies on dantrolene as a treatment for depression and anxiety. Control groups received no pretreatment. Dantrolene nanoparticles were freshly prepared before each administration, and an intranasal dantrolene nanoparticle stock solution was made at 5 mg/ml. The Ryanodex formulation vehicle was made fresh and contained all inactive ingredients in Ryanodex [48, 49]. At the end of the 4-week pretreatment period, mice were treated with a one-time intraperitoneal injection of lipopolysaccharides (LPS, 5 mg/kg). Mice in the control group, which did not receive pretreatment, were administered LPS (LPS, 5 mg/kg) once as well. The sham control group received no pretreatment or treatment. 24 h following the one-time LPS treatment, behavioral tests for depression and anxiety behaviors were performed on all mice, with the following order tests ranked for increasing stress: OPT, EPMT, TST, and FST. Both the animal behavior investigators and the data analysts were unaware of the animal grouping information, which effectively avoided investigator bias. Afterwards, all mice were euthanized, and the blood and brain were harvested to examine inflammation, programmed cell death via pyroptosis, and synapse destruction.

### Intranasal dantrolene nanoparticle or vehicle administration

Dantrolene (Sigma, St Louis, MO) was dissolved in the Ryanodex Formulation Vehicle (RFV: 125 mg mannitol, 25 mg polysorbate 80, 4 mg povidone K12 in 5 mL of sterile water, with the pH adjusted to 10.3), similar to our previous publications [49, 50]. For intranasal administration, the final concentration of dantrolene was 5 mg/mL. Mice were held and fixed by the scruff of their necks with one hand. On the other hand, mice were administered 1 μL/gram body weight of dantrolene nanoparticles in the RFV, or the empty vehicle (e.g., a mouse weighing 20 g would be given 20 μL of solution, equivalent to dantrolene dosing at 5 mg/kg). The solution was slowly delivered directly into the mouse’s nose. Care was taken to ensure that mice were minimally stressed and that the solution remained in the nasal cavity and did not enter the stomach or lungs.

### Forced swimming test (FST)

Depression behavior was assessed using the FST, as previously described in other publications [51]. In each experimental condition, mice were placed in a clear glass tank (25 cm height, 10 cm diameter, 15 cm water depth), with the water’s temperature set to 23 ± 2 °C. The total test duration was 6 min: 2 min for adaptation, followed by 4 min of measured and recorded immobility time. Immobility occurred when the mouse ceased floundering and floated passively in an equilibrium state. The greater the immobility time, the more severe the depression behavior was considered.

### Tail suspension test (TST)

Depression behavior was assessed using the tail suspension test, as described previously, with the following modifications [52]. Specially manufactured tail suspension boxes made of cardboard, with dimensions of 42 cm (height) x 18 cm (width) x 30 cm (depth) were utilized. To prevent mice from observing or interacting with each other, each mouse was suspended within its own three-walled rectangular box. Animals were acclimated to the testing environment for at least one hour prior to the test. Each mouse was suspended in the middle of the box. The width and depth of the box were sufficiently sized to prevent the mouse from contacting the walls. In this setting, the approximate distance between the mouse’s nose and the apparatus floor was 20-25 cm. A plastic suspension bar, with dimensions of 50 cm (height) x 40 cm (depth), was positioned at the top of the box and used to suspend the tail of each mouse. At the bottom of each box, paper towels were placed to collect feces and urine. A dark grey box was used for albino-colored mice, while a white colored box was used for mice of other coat colors. Prior to evaluation, the tail of each mouse was securely attached to the suspension bar, which was able to withstand the mouse’s weight. A video camera was placed in position to record the TST session. The total duration of the test was 6 min. The paper towel was replaced after each trial. After all sessions were finished, Any-maze software (Stoelting, USA) was used to analyze collected data.

During behavioral analysis, the mobility time of each mouse was measured and subtracted from 360 s, yielding the immobility time. The greater the immobility time, the more severe the depressive behavior was considered.

### Open field test (OFT)

The OFT was performed to evaluate anxiety behavior as described previously in our other publications [53]. Each mouse was individually placed into the OFT apparatus (dimensions of 44 x 44 x 44 cm^3^), facing the wall. A camera was placed above the apparatus in dim light and locomotor activity was recorded for 6 min. The anxiety behavior of each mouse was measured using the total distance traveled, number of center entries, immobility time, and time spent in the central zone. After each test, the apparatus was cleaned with 75% ethanol. Any-maze software (Stoelting USA) was used to analyze collected data. The less the central zone distance and mean speed, the greater the anxiety behavior was considered. The greater the immobility time, the more severe the anxiety behavior was considered.

### Elevated plus maze test (EPMT)

Anxiety behavior was assessed using EPMT, as described previously in our other publications [54]. Mice were placed in the center of an elevated plus-maze (dimensions of 33 cm (length) x 5 cm (width) x 25 cm (height) for the closed arms), under dim lighting. Behavior was observed and recorded for 5 min. Any-maze software (Stoelting USA) was used to analyze the collected data. Time spent in the closed arms, time spent in the open arms, and the number of explorations of open arms were measured and recorded. The greater the time spent in the closed arms, the more severe the anxiety behavior was considered.

### Euthanasia and tissue collection

Mice were euthanized after completion of all behavioral tests. As described previously [49], mice were deeply anesthetized with 2–4% isoflurane delivered through a nose cone, with the concentration adjusted according to toe pinch response. The skin of each mouse was prepared, and an incision was made to open the chest and expose the heart. Blood was collected from the heart using a 27 G needle and syringe for the serum study. Blood was centrifuged at 1400 rpm at 4 °C for 30 min, and the supernatant was collected and frozen at –80 °C. The mice were euthanized by transcardial perfusion and exsanguinated with cold phosphate-buffered saline.

### Measurements of serum concentration of IL-1β and IL-18

Following company instructions, serum IL-1β and IL-18 cytokines were measured using an ELISA kit. as described previously for the measurement of S100β [55, 56]. All reagents and serum samples were thawed to room temperature (18–25 °C) before use. All standards and samples were run at least in duplicate. 100 µL of each standard and sample was added to the appropriate wells. After the wells were covered, the standards were incubated for 2.5 h at room temperature, while the samples were incubated overnight at 4 °C with gentle shaking. The solution was discarded and washed 4 times with 1X Wash Solution. Each well was then filled with Wash Buffer (300 µL) using a multi-channel pipette or auto washer After the last wash, remaining Wash Buffer was removed by aspirating or decanting. The plate was inverted and blotted against clean paper towels. 100 µL of 1X prepared Detection Antibody was then added to each well. Wells were covered and incubated for 1 h at room temperature with gentle shaking. The solution was then discarded and the procedure outlined in step 3 was repeated. 100 µL of the prepared Streptavidin solution was added to each well. The wells were covered and incubated for 45 min at room temperature with gentle shaking. The solution was discarded, and the wash was repeated as outlined in step 3. 100 µL of TMB One-Step Substrate Reagent (Item H) was then added to each well. The wells were covered and incubated for 30 min at room temperature in the dark with gentle shaking. 50 µL of Stop Solution (Item I) was added to each well, whereupon the absorbance was then immediately read at 450 nm by a synergy H1 plate reader (Agilent, CA, USA). The mouse IL-1β and IL-18 ELISA Kits were purchased from SIGMA(RAB0275-1KT/RAB08100-1KT).

### Immunoblotting (Western Blot)

As previously described in our other publications [57], whole brain tissues were extracted by homogenization using cold RIPA buffer (#9806S, Cell Technology, USA), supplemented with protease inhibitor cocktails (P8340 Roche). Brain homogenates were rocked at 4 °C for 90 min and then centrifuged at 14,000 rpm (Brushless Microcentrifuge, Denville 260D) for 20 min at 4 °C to remove cell debris. After collecting the supernatant, protein concentration was measured using a BCA protein assay kit (Pierce, Rockford, IL 61101 USA). To briefly describe, equal amounts of protein (20 µg/lane) were loaded onto 4–20% gel electrophoresis of mini-protein TGX precast (Cat. #4561094, BIO-RAD) and transferred to polyvinylidene difluoride (PVDF) membranes (Immobilon-P, MERK Millipore, Ireland) using a wet electrotransfer system (BIO-RAD, USA). The PVDF membrane was blocked with 5% BSA (Sigma-Aldrich) for 1 h and subsequently incubated overnight at 4 °C with primary antibodies targeting NLRP3, cleaved caspase-1, GSDMD-NT, IL-1β, IL-18, PSD95, Synapsin-1, and GAPDH (Table 1). After incubation with HRP-conjugated secondary antibodies (anti-mouse IgG1 and anti-rabbit IgG), the membranes were washed three times for 10 min each with TBST (Tris-buffered saline containing 0.2% Tween-20). Protein bands were detected using ECL Western Blotting Detection Reagents (Cytiva, Amersham, UK) and quantified with ImageJ software (NIH, Bethesda, MD, USA) by two independent investigators. The results were then averaged for each mouse.

**Table 1:** Antibodies used for Western Blot.

	Cat. and Manufacture	Dilution
Primary antibody		
Anti- NLRP3	68102-1-Ig; Proteintech	1:2 000
Anti-Human procaspase-1/ cleaved caspase-1 p20	#2225S, Cell signaling Technology,USA	1:1 000
Anti- Cleaved GSDMD	#36425S; Cell signaling Technology, USA	1:1 000
Anti-IL-1β	31202S, Cell signaling Technology, USA	1:1 000
Anti-IL-18	10663-1-AP; Proteintech	1:3 000
Anti-PSD95	#2507; Cell signaling Technology, USA	1:1 000
Anti-Synapsin-1	#5297; Cell signaling Technology, USA	1:1 000
GAPDH	MA5–15738, Thermo Fisher Scientific	1:10 000
Secondary antibody		
Anti-Mouse IgG1; HRP -linked	# PA1-74421, Thermo Fisher Scientific	1:10 000
Anti-rabbit IgG, HRP-linked	#7074; Cell signaling Technology, USA	1:1 000

### Statistical analysis

All data were represented as mean ± one standard deviation (Means ± SD). Statistical analysis was conducted using GraphPad Prism (Version 9.3.1, CA, USA). Comparisons of more than two groups were conducted using one-way ANOVA with Tukey’s multiple comparison test. The sample size (N) was estimated to be around 15 mice per group for adequate power analysis based on our previous similar studies [49, 58]. Normal distribution tests were performed for all data. Two-sided p-values were reported. P < 0.05 was considered statistically significant.

## Results

### Intranasal dantrolene nanoparticle pretreatment significantly inhibited LPS-induced helplessness and anxiety-related behaviors

**Fig. 1:**
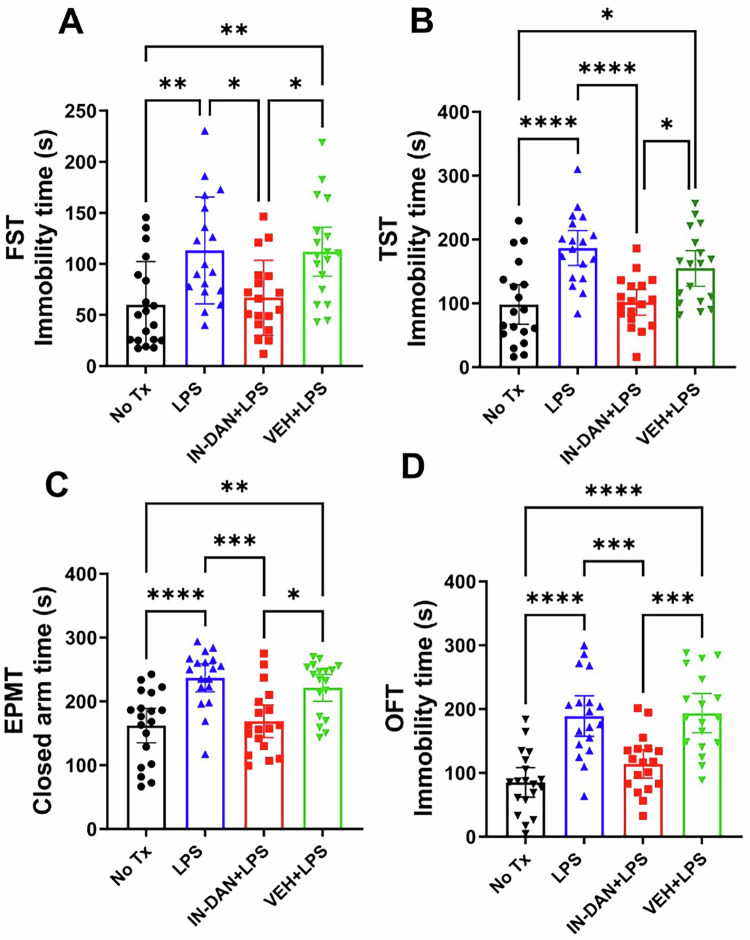
Intranasal dantrolene nanoparticles significantly reduced LPS-induced acute helplessness and anxiety-related behaviors. Adult B6SJLF1/J mice were treated with intranasal dantrolene nanoparticles (IN-DAN) or vehicle (VEH) for 28 days, followed by a single i.p. injection of lipopolysaccharide (LPS) (IN-DAN + LPS). Control mice either had no treatment (No Tx), only LPS (LPS), or VEH + LPS. The forced swim test (FST, **A**), tail suspension test (TST, **B**), elevated plus maze (EPMT, **C**) and open field test (OFT, **D**) were performed 24 h after the LPS injection. Increased mobility time indicates helplessness or anxiety-related behaviors (**A,** **B,** **D**). Increased time in the closed arm indicates anxiety (**C**). N = 18-19 mice, Mean±95%CI, One-Way ANOVA followed by Tukey post hoc test. *P < 0.05, **P < 0.01, ****P < 0.0001.

The FST and TST are commonly used to evaluate helplessness and depression behaviors in mice, as well as to compare drug efficacy and side effects [23, 59, 60]. In both the FST (Fig. 1A) and the TST (Fig. 1B), LPS-induced experimental groups exhibited significantly higher immobility times (indicating helplessness or depressive behaviors), increasing 89% (60.11 ± 42.24 vs. 113.3 ± 52.34) and 90% (98.16 ± 63.99 vs. 186.6 ± 54.98), respectively. However, intranasal dantrolene nanoparticle pretreatment robustly inhibited these increases in immobility time, diminishing by 41% in the FST (113.3 ± 52.34 vs. 66.88 ± 36.83) and 46% in the TST (186.6 ± 54.98 vs. 101.5 ± 40.22). In contrast, the intranasal dantrolene nanoparticle vehicle control condition did not demonstrate these same inhibitory effects on helpless behaviors. The elevated plus maze test (EPMT) and open field test (OPT) are commonly used to evaluate anxiety-related behaviors in mice and to test drug efficacy in treating anxiety behaviors [23, 61, 62]. In both the EPMT (Fig. 1C) and OFT (Fig. 1D), LPS administration significantly increased depression and anxiety behaviors, as indicated by a 46% increase in the time spent in the closed arms of the EPMT (162.2 ± 56.03 vs. 237.0 ± 44.18) and a 122% increase in observed immobility time in the OFT (85.06 ± 47.89 vs. 188.9 ± 63.97). Intranasal pretreatment with dantrolene nanoparticles significantly attenuated these behavioral alterations, reducing time in the closed arms by 29% (237.0 ± 44.18 vs. 168.8 ± 51.44) and immobility time by 40% (188.9 ± 63.97 vs. 113.9 ± 44.61). Conversely, the vehicle-treated controls did not exhibit these protective effects. In the open field test (Supplementary Fig. 2), reduced central zone distance and locomotor speed reflected increased anxiety behavior. LPS administration significantly decreased mean speed relative to untreated controls but did not alter central zone distance. Intranasal dantrolene nanoparticle pretreatment partially reversed the LPS-induced reduction in mean speed and central zone distance.

### Intranasal dantrolene nanoparticle pretreatment significantly inhibited LPS-induced helplessness and anxiety behaviors primarily in female mice

**Fig. 2:**
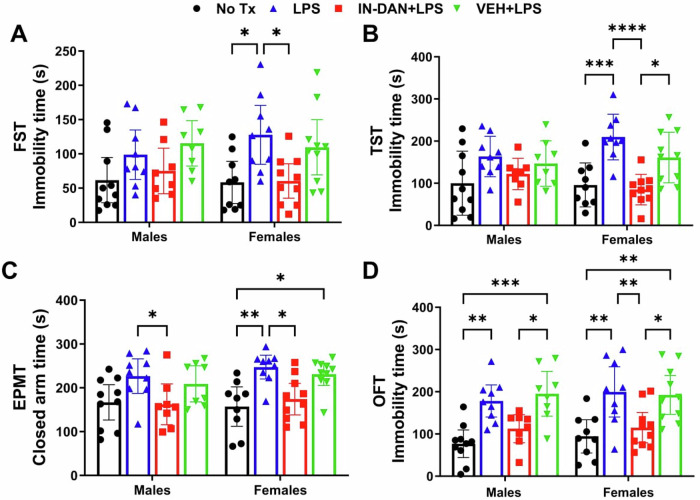
Intranasal dantrolene nanoparticles reduced LPS-induced acute helplessness and anxiety-related behaviors in more female than male mice. The sex of the adult B6SJLF1/J mice, pretreated with intranasal dantrolene nanoparticles (IN-DAN) or vehicle (VEH) for 28 days, followed by a single i.p. injection of LPS (IN-DAN + LPS), was examined. No treatment controls (No Tx), LPS only (LPS), or VEH + LPS. The forced swimming test (FST, **A**), tail suspension test (TST, **B**), elevated plus maze (EPMT, **C**), and open field test (OFT, **D**) were performed 24 h after the LPS injection. N = 8-10 mice, Mean±95%CI, Two-Way ANOVA followed by Tukey post hoc test. *P < 0.05,**P < 0.01, ****P < 0.0001.

Sex differences in the neuroprotective effects of intranasal dantrolene nanoparticles on helplessness and anxiety behaviors were then evaluated. As illustrated in Fig. 2, dantrolene nanoparticle treatment, but not the vehicle control, significantly reduced helplessness behaviors in female mice, as measured by the FST (Fig. 2A) and TST (Fig. 2B); no such effects were observed in males. For anxiety behaviors, dantrolene nanoparticles attenuated LPS-induced anxiety in males as assessed by the EPMT (Fig. 2C), but the most robust effects were observed in females, where reductions were evident in both the EPMT (Fig. 2C) and OFT (Fig. 2D).

### Intranasal dantrolene nanoparticles significantly inhibited LPS-induced pathological elevation of pyroptosis-related inflammation cytokines in blood and brains

**Fig. 3:**
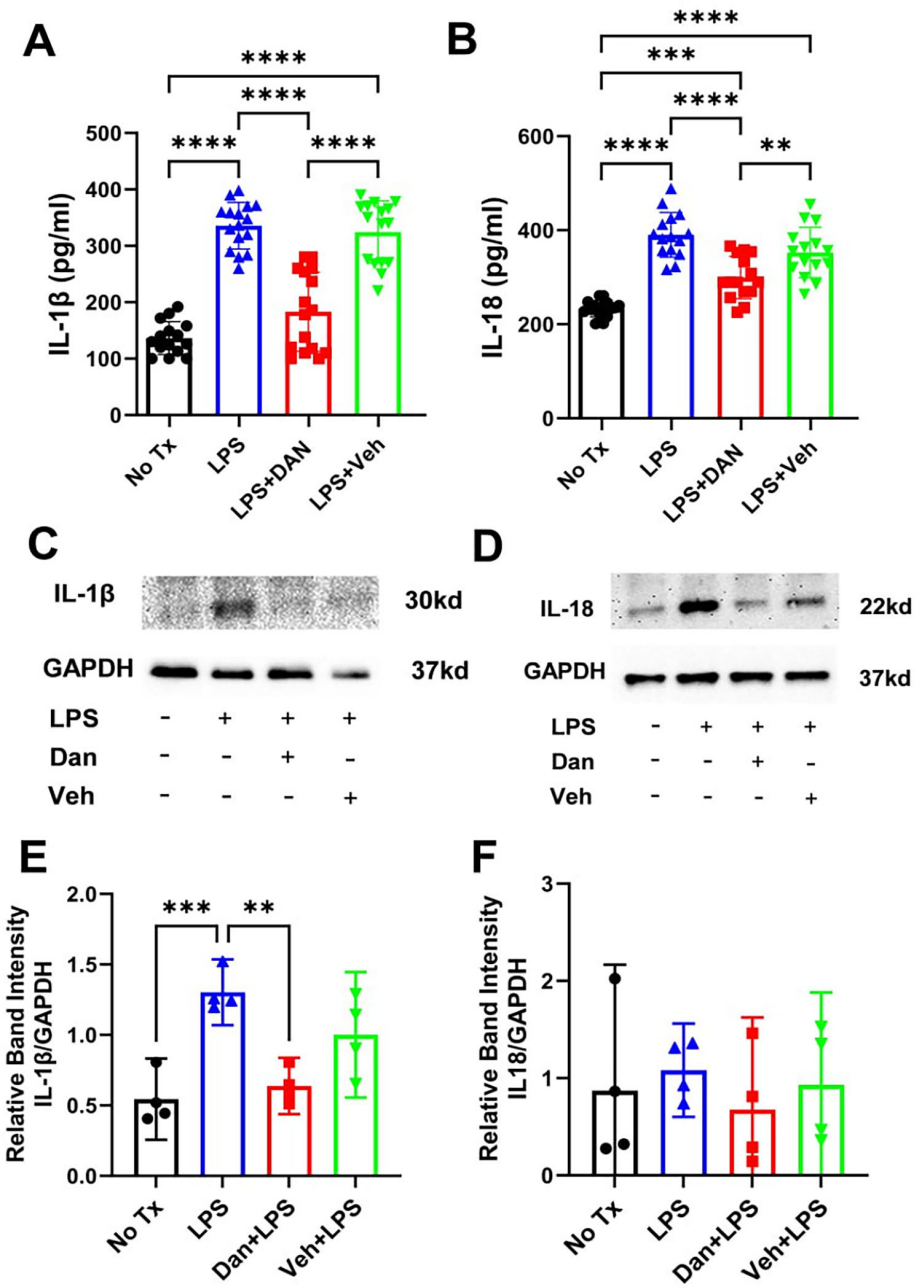
Intranasal dantrolene significantly inhibited lipopolysaccharide-induced pathological elevation of IL-18 and IL-1β cytokines in the blood and brains. B6SJLF1/J adult mice were pretreated with intranasal dantrolene nanoparticles (Dan, 5 mg/kg) or vehicle (Veh) control daily, Monday to Friday, for continuous 4 weeks. Mice were then treated with one-time IP injection of lipopolysaccharide (LPS, 5 mg/kg). Mice in control (No Tx) group received no treatments. IL1β in blood (**A**) and brain (**C,** **E**) and IL18 in blood (**B**) and brains (**D,** **F**) were measured using ELISA assay kit (**A,** **B**) or immunoblotting (**C**-**F**)., N = 18-20 mice for blood measurement (**A,** **B**) and N = 4 brains for immunoblotting (**C**-**F**), Means±95% CI, One-Way ANOVA followed by Tukey post hoc test. **P < 0.01, ****P < 0.0001, respectively.

Compared with untreated controls, a single intraperitoneal injection of LPS (5 mg/kg) significantly increased blood IL-1β concentrations by 146% (136.4 ± 29.31 vs. 335.6 ± 41.12; Fig. 3A) and IL-18 concentrations by 67% (233.1 ± 17.20 vs. 390.2 ± 47.65; Fig. 3B). Intranasal dantrolene nanoparticle treatment, but not the vehicle control, significantly attenuated these increases, reducing IL-1β levels by 46% (335.6 ± 41.12 vs. 183.2 ± 70.05) and IL-18 levels by 23% (390.2 ± 47.65 vs. 299.2 ± 45.18; Fig. 3B). Moreover, dantrolene nanoparticles significantly suppressed LPS-induced elevations of IL-1β and IL-18 protein levels in both the blood (Fig. 3A, B) and brain tissue (Fig. 3C, D; Supplementary Figs. 3 and 4).

### Intranasal dantrolene nanoparticles modulate LPS-induced programmed cell death by pyroptosis in the mouse brain

**Fig. 4:**
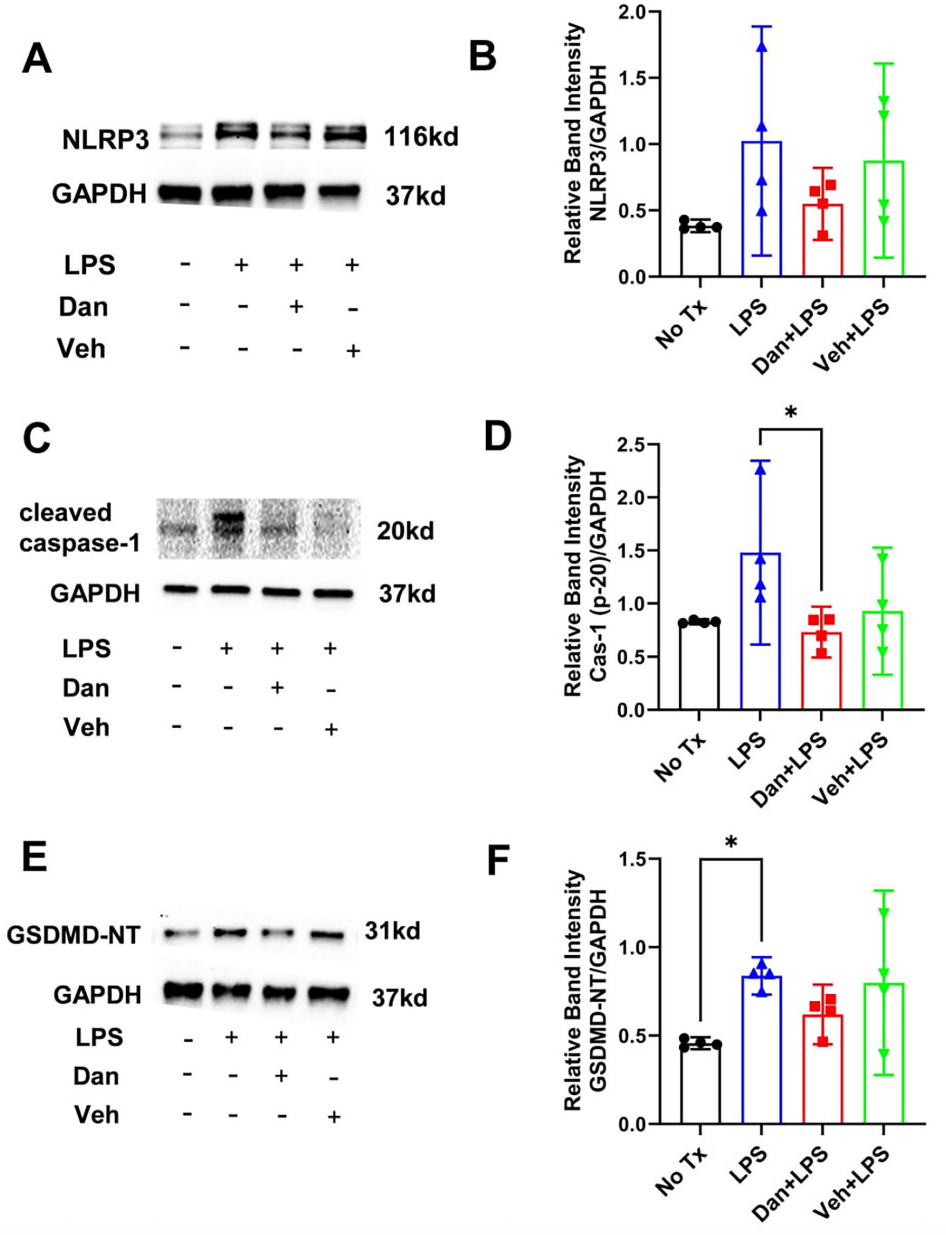
Effects of intranasal dantrolene nanoparticles on lipopolysaccharide-induced programmed cell death by pyroptosis in the brains. B6SJLF1/J adult mice (5–10 months old) were pretreated with intranasal dantrolene nanoparticles (Dan, 5 mg/kg) or vehicle (Veh) daily, Monday to Friday, for continuous 4 weeks. Mice were then treated with a one-time IP injection of lipopolysaccharide (LPS, 5 mg/kg). Mice in control (No Tx) group received no treatments. Representative western blot (**A,** **C,** **E**) and statistical analysis (**B,** **D,** **F**) determined the protein levels of critical regulatory proteins of pyroptosis pathway (**A,** **B**, NLRP3; **C,** **D**, Active caspase-1 (P-20), **E,** **F**, N terminal GSDMD (GSDMD-NT) in brains. N = 4 different mice brain in each group. GAPDH as loading control. Means±95%CI, One-Way ANOVA followed by Tukey post hoc test. * P < 0.05.

Relative to untreated controls, LPS administration elevated expression of pyroptosis-related proteins (NLRP3, caspase-1, and N-terminal GSDMD) in mouse brain tissue, with a significant increase in GSDMD. Intranasal dantrolene nanoparticles significantly inhibited the LPS-induced elevation of caspase-1 (P20), a marker of programmed cell death through pyroptosis and apoptosis^65^,^66^ (Fig. 4C, D), and reduced pyroptosis pathway activation, with a significant effect on cleaved caspase-1 (Fig. 4).

### Intranasal dantrolene nanoparticles significantly inhibited LPS-induced synapse protein loss in mouse brains

**Fig. 5:**
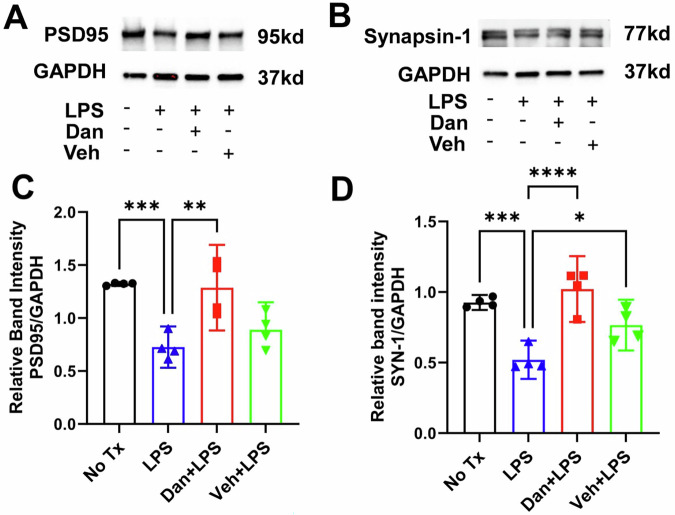
Intranasal dantrolene nanoparticles significantly inhibited lipopolysaccharide-induced synapse protein loss in brains. B6SJLF1/J adult mice were pretreated with intranasal dantrolene nanoparticles (Dan, 5 mg/kg) or vehicle (Veh) daily, Monday to Friday, for continuous 4 weeks. Mice were then treated with one-time IP injection of lipopolysaccharide (LPS, 5 mg/kg). Mice in control (No Tx) group received no treatments. Western blot measured changes of synapse proteins of PSD-95 (**A,** **C**) and Synapsin-1 (**B,** **D**) in the brains. GAPDH as loading control. N = 4 different mice in each group, Means±95%CI, One-Way ANOVA followed by Tukey post hoc test. *P < 0.05, **P < 0.01, ***P < 0.001, ****P < 0.0001, respectively.

Compared with untreated controls, LPS administration significantly reduced synaptic protein levels of PSD-95 by 45% (1.322 ± 0.0118 vs. 0.726 ± 0.123; Fig. 5A, C) and synapsin-1 by 44% (0.926 ± 0.0335 vs. 0.520 ± 0.0851; Fig. 5B, D) in mouse brains. Four weeks of intranasal pretreatment with dantrolene nanoparticles, but not the vehicle control, significantly attenuated these reductions, restoring PSD-95 by 77% (0.726 ± 0.123 vs. 1.286 ± 0.2538; Fig. 5A, C) and synapsin-1 by 76% (0.520 ± 0.0851 vs. 1.021 ± 0.1467; Fig. 5B, D).

## Discussion

There is an urgent need for novel therapeutic interventions to prevent or treat MDD [3, 63] and anxiety [5] psychiatric disorders, especially in cases of drug-resistant or recurrent depression and anxiety [5, 12, 64]. In this study, intranasal dantrolene nanoparticles were shown to ameliorate helplessness and anxiety behaviors in adult mice. This effect was evidenced by the inhibition of pathological elevations of inflammatory cytokine levels (IL-1β and IL-18) in the blood and the pyroptosis biomarker caspase-1 (P20), as well as the loss of synaptic proteins in the brain. Considering that upstream Ca^2+^ dysregulation plays a critical role in the pathology of depression and anxiety, supported by the clinical efficacy of ketamine and esketamine in treatment-resistant cases [10, 12], these findings suggest that dantrolene, or related compounds, may represent a new class of therapeutics. These would be aimed at correcting upstream Ca²⁺ dysregulation and its downstream consequences, including pathological inflammation and pyroptosis, in psychiatric disorders.

Increasing studies suggest that inflammation plays a critical role in the pathophysiology of MDD and anxiety, making it an attractive therapeutic target [65–68]. Intracellular Ca^2+^ dysregulation has been proposed as an upstream driver of mitochondrial dysfunction, oxidative stress, and inflammation in MDD [21, 69, 70] and anxiety [34, 71]. Notably, inhibition of Ca²⁺ dysregulation via suppression of RyR overactivation has been shown to reduce inflammation in Gulf War illness, a condition associated with depression and dementia [24]. Dantrolene, a clinically available RyR antagonist, has previously demonstrated anti-inflammatory effects in diabetes [72, 73], sepsis [74, 75], and COVID-19 infection [76, 77]. However, its ability to inhibit Ca²⁺ dysregulation and related inflammation in the context of depression and anxiety had not been investigated prior to this study.

Several animal models have been developed to study depression and anxiety behaviors, based on proposed neurobiological mechanisms such as altered neurotransmission, hypothalamic–pituitary–adrenal (HPA) axis dysregulation under chronic stress, inflammation, reduced neuroplasticity, and network dysfunction [78–80]. Among these, induction of inflammation with lipopolysaccharide (LPS) has become a widely used approach to model depression and anxiety and to evaluate the efficacy of potential treatments [33, 69, 81–84]. Intraperitoneal (IP) administration is the most common method, given its ease and reproducibility, though effective dosing and regimens vary. A single high dose of LPS (5 mg/kg) has been shown to reliably induce inflammation and depressive behaviors [69]. Consistent with these findings, our study confirmed that one-time IP LPS administration (5 mg/kg) produced robust and reproducible inflammation, as well as depression and anxiety behaviors in adult mice, providing a suitable model for evaluating the therapeutic efficacy of dantrolene.

Using this model, we demonstrated that intranasal dantrolene nanoparticle pretreatment robustly attenuated LPS-induced inflammation and associated helplessness and anxiety behaviors. In addition to its previously reported protective effects against memory loss in various AD animal models [49, 85–87], intranasal dantrolene nanoparticles also significantly inhibited neuroinflammation, pyroptosis-related cytokines (IL-1β and IL-18), and synaptic protein loss, highlighting a role for inflammation and pyroptosis in both cognitive dysfunction and psychiatric disorders. These findings suggest that targeting inflammation may represent a unifying therapeutic strategy for treating both memory impairment and psychiatric symptoms in conditions such as Alzheimer’s disease. Importantly, our data indicates that intranasal dantrolene nanoparticles could be developed as a novel therapeutic approach for major depressive disorder (MDD) and anxiety, potentially through inhibition of pathological inflammation.

MDD affects women at twice the rate of men [88, 89]. Proposed mechanisms underlying this sex difference include the influence of estrogen [90], alterations in serotonin and tryptophan metabolism [91], and differential stress responses [89]. An important finding in this study is that intranasal dantrolene nanoparticles were more effective in ameliorating helplessness and anxiety behaviors in female mice than in male mice. This observation aligns with the higher prevalence of MDD in females and suggests that dantrolene may target key pathophysiological processes contributing to sex differences in depression and anxiety. The more pronounced inhibition of inflammation observed in female mice further supports the possibility that dantrolene acts by suppressing upstream Ca²⁺ dysregulation, thereby mitigating downstream pathological processes, including inflammation, pyroptosis, and synaptic protein loss. Although the precise mechanisms remain to be elucidated, these findings highlight a potential sex-specific therapeutic benefit of intranasal dantrolene nanoparticles.

Intranasal drug delivery, particularly in a nanoparticle formulation, offers a promising strategy to bypass the blood-brain barrier (BBB) and enhance central nervous system penetration with minimal peripheral toxicity [92–94]. Our previous work demonstrated that intranasal dantrolene nanoparticles achieve greater therapeutic efficacy in the brain compared with oral and subcutaneous forms of administration, with minimal or no side effects following chronic use [49, 50, 58]. Notably, intranasal dantrolene nanoparticles significantly ameliorated memory loss in 5XFAD mice, supporting their potential as a disease-modifying therapy [49]. RyR overactivation and the resulting Ca^2+^ dysregulation have been identified as upstream pathological events that drive mitochondrial dysfunction [95], oxidative stress [96], pathological inflammation [97], and pyroptotic neuronal death [98]. These processes contribute to the development of depression and anxiety disorders [30, 68, 99]. In the present study, we demonstrated that inhibition of RyR overactivation with dantrolene robustly attenuated pathological inflammation, prevented synaptic protein loss, and ameliorated depressive and anxiety behaviors in mice. These findings reinforce the concept that upstream Ca²⁺ dysregulation and downstream inflammation and synaptic damage are critical drivers of psychiatric pathology. Similar to esketamine [18, 100], intranasal dantrolene nanoparticles hold promise as a potential disease-modifying treatment for depression and anxiety and warrant further investigation in clinical studies.

Since depression and anxiety are chronic disorders, effective treatments must be suitable for long-term use with minimal side effects or organ toxicity. A key advantage of utilizing intranasal dantrolene nanoparticles, compared with oral or intravenous administration, is their significant increase in the brain-to-blood concentration ratio of dantrolene [49, 50], particularly in aged mice [58]. This enhanced CNS delivery improves therapeutic efficacy while reducing systemic exposure and minimizing adverse effects [49]. In our previous studies, chronic intranasal administration of dantrolene nanoparticles for up to 10 months in adult 5XFAD mice did not cause detectable side effects on nasal structure or olfactory function [49, 101], liver structure [49] or function, or muscle function [48, 49, 85]. These findings suggest that intranasal dantrolene nanoparticles are well-tolerated with chronic use in animal models. Nonetheless, further studies are needed to evaluate the long-term safety and tolerability of chronic dantrolene treatment in human patients.

This study has the following limitations: 1) The mice used were 5–10 months old, an age range considered adult; however, future studies should examine different age groups, particularly aged mice, given the high prevalence of psychiatric disorders in older populations. The use of littermates within each experimental group may bias the observations in behavioral testing. 2) We were unable to measure cytosolic versus mitochondrial Ca²⁺ levels in brain tissue due to technical challenges. Nevertheless, prior studies have demonstrated that dantrolene inhibits LPS- or AD-related gene mutation-induced RyR overactivation and Ca²⁺ dysregulation in cell culture models [102, 103]. 3) We did not measure reactive oxygen species (ROS) concentrations in the brain, which are typically upstream drivers of NLRP3 inflammasome activation [104]. 4) Although intranasal dantrolene nanoparticles robustly and significantly inhibited LPS-induced pathological inflammation, as well as depression and anxiety behaviors, their effects on programmed cell death by pyroptosis could not be consistently confirmed, underscoring the need for further mechanistic studies of dantrolene’s neuroprotective actions. 5) We did not investigate dose-response effects of dantrolene on depression and anxiety behaviors. Because physiological Ca²⁺ release from the ER is essential for cellular function, excessive inhibition could be detrimental. Determining the optimal dose of intranasal dantrolene nanoparticles for psychiatric disorders is therefore critical before translation to clinical use. 6) Biomarker measurements were performed in whole-brain tissue rather than in specific regions, which may have masked region-specific alterations and reduced sensitivity to detect localized changes relevant to disease processes.

In conclusion, this study demonstrates that intranasal dantrolene nanoparticles significantly ameliorated inflammation-induced depression and anxiety behaviors in adult mice. These neuroprotective effects were associated with suppression of pathological cytokine elevation in the blood and preservation of synaptic proteins in the brain. Taken together, our findings highlight the potential of intranasal dantrolene nanoparticles as a novel therapeutic strategy for psychiatric disorders, warranting further preclinical and clinical investigation.

## Supplementary information

Supplementary Information

## Data Availability

Data available upon request.
