# Supplementary material for: Intranasal dantrolene nanoparticles inhibit lipopolysaccharide-induced depression and anxiety behavior in mice
Source: Transl Psychiatry. 2026 Feb 3;16:104. doi: 10.1038/s41398-026-03816-x (PMC12923542; doi:10.1038/s41398-026-03816-x)
Supplement: Supplementary file 1 — Supplementary Information [file 41398_2026_3816_MOESM1_ESM.pdf]

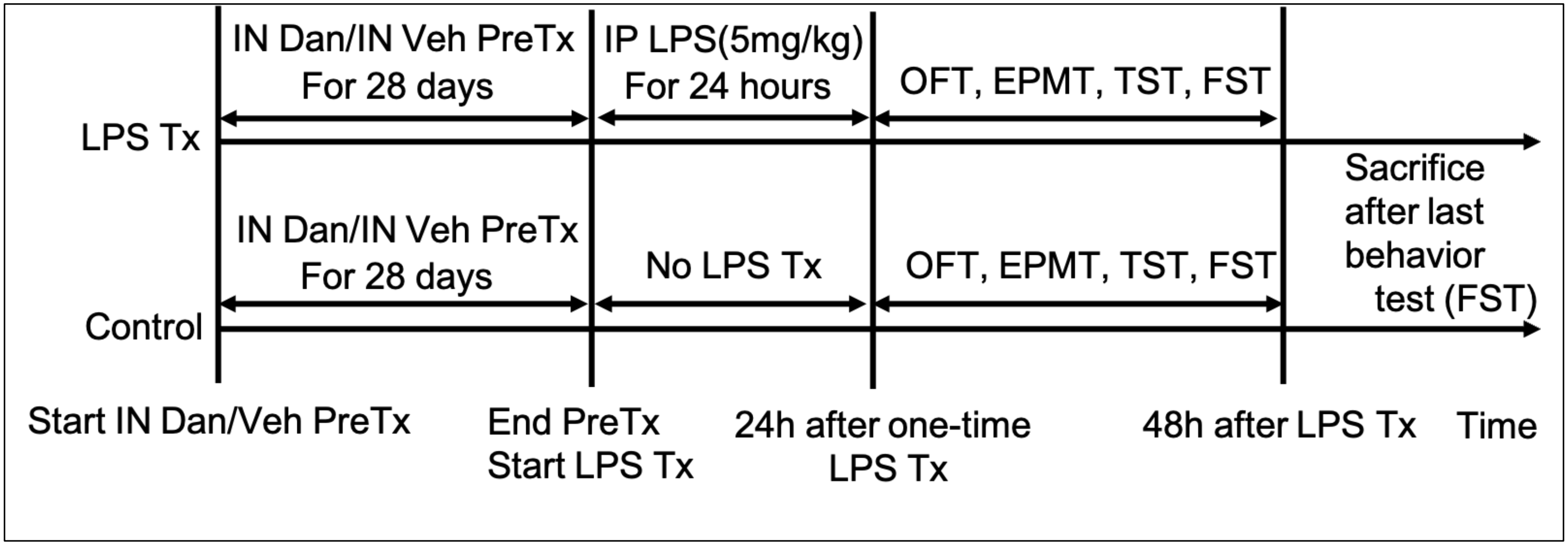

Supplemental figure 1. Experimental Design. Adult B6SJLF1/J mice were pretreated with intranasal dantrolene nanoparticles in Ryanodex formulation (DNRF, 5mg/kg) or vehicle (Veh) for 4 weeks. Control group received no treatments. Mice were then treated with intraperitoneal (IP) injection of lipopolysaccharide (LPS, 5mg/kg) for one time. Depression and anxiety behavior tests were performed at 24 hours after one-time IP LPS injection. PreTX: Pretreatment, Tx: Treatment, IN: Intranasal, Dan: Dantrolene, OFT: Open Field Test, EPMT: Elevated Plus Maze Test, TST: Tail Suspension Test, FST: Forced Swimming Test

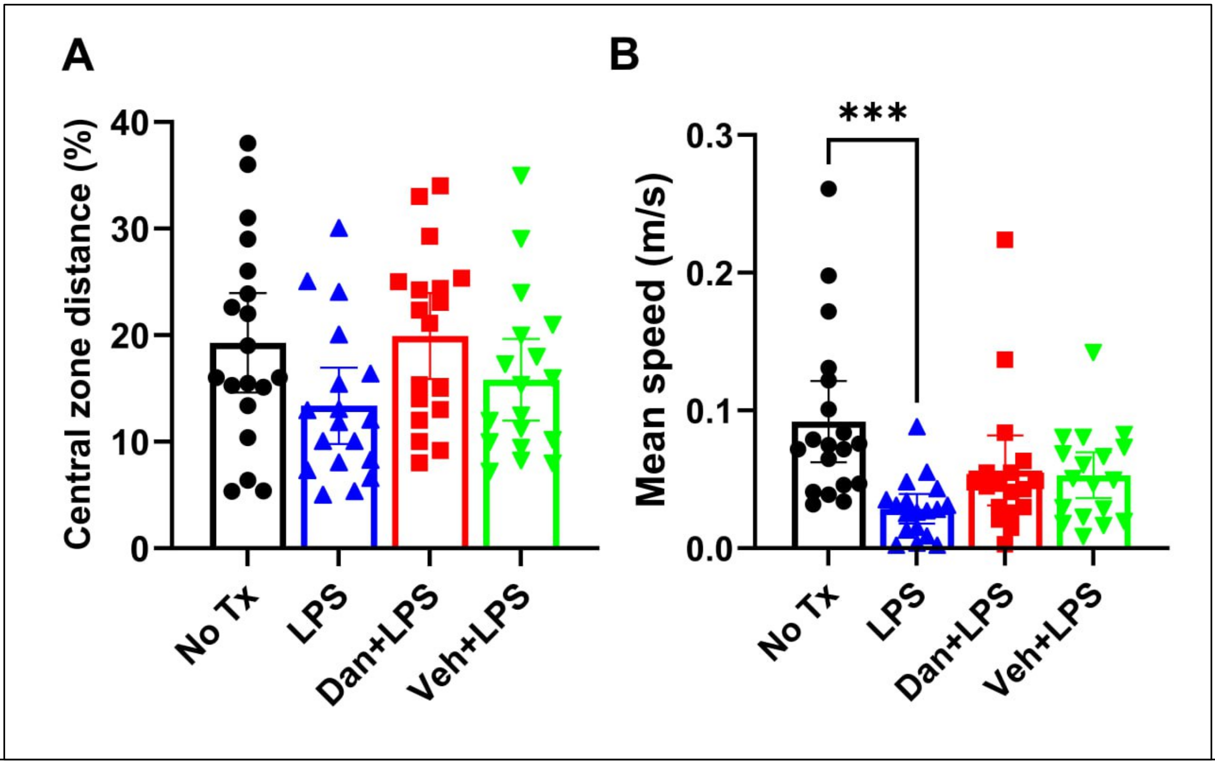

Supplemental figure 2. Effects of intranasal dantrolene nanoparticles on lipopolysaccharide-induced anxiety behavior in adult mice. B6SJLF1/J adult mice (5-10 months old) were pretreated with intranasal dantrolene nanoparticles (Dan, 5mg/kg) or vehicle (Veh) control daily, Monday to Friday, for continuous 4 weeks. Mice were then treated with one-time IP injection of lipopolysaccharide (LPS, 5 mg/kg). Mice in control (No Tx) group received no treatments. Open field test (OFT) was performed at 24 hours after one-time IP LPS injection. The less central zone distance or less mean speed, the more anxiety behavior for OFT. N=18-20 mice. Means±95%CI, One-Way ANOVA followed by Tukey post hoc test. ***P<0.001.

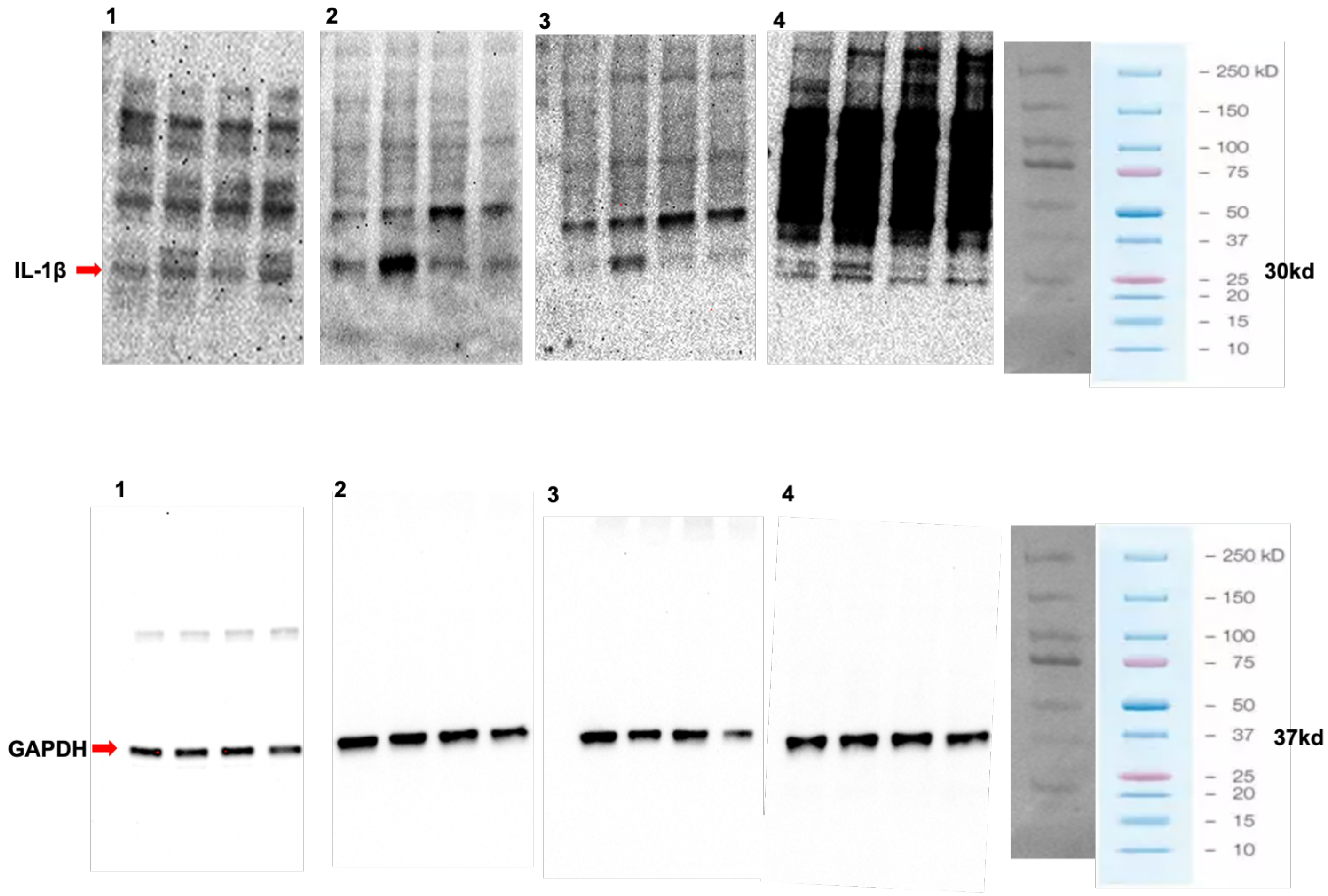

Supplemental figure 3. The uncropped images of the original western blots in figure 3C. Repeated from 4 individual groups.

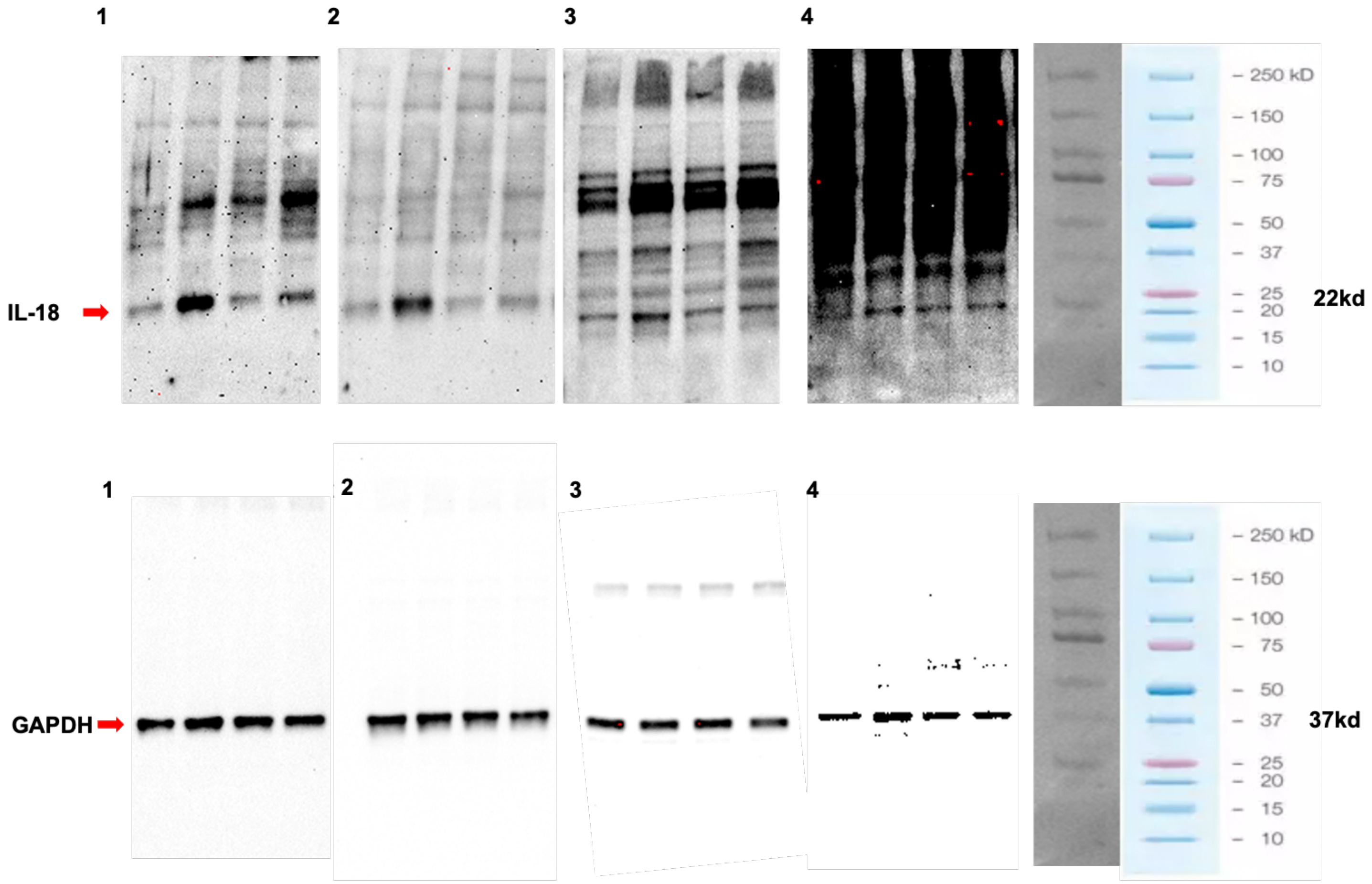

Supplemental figure 4 The uncropped images of the original western blots in figure 3D. Repeated from 4 individual groups.

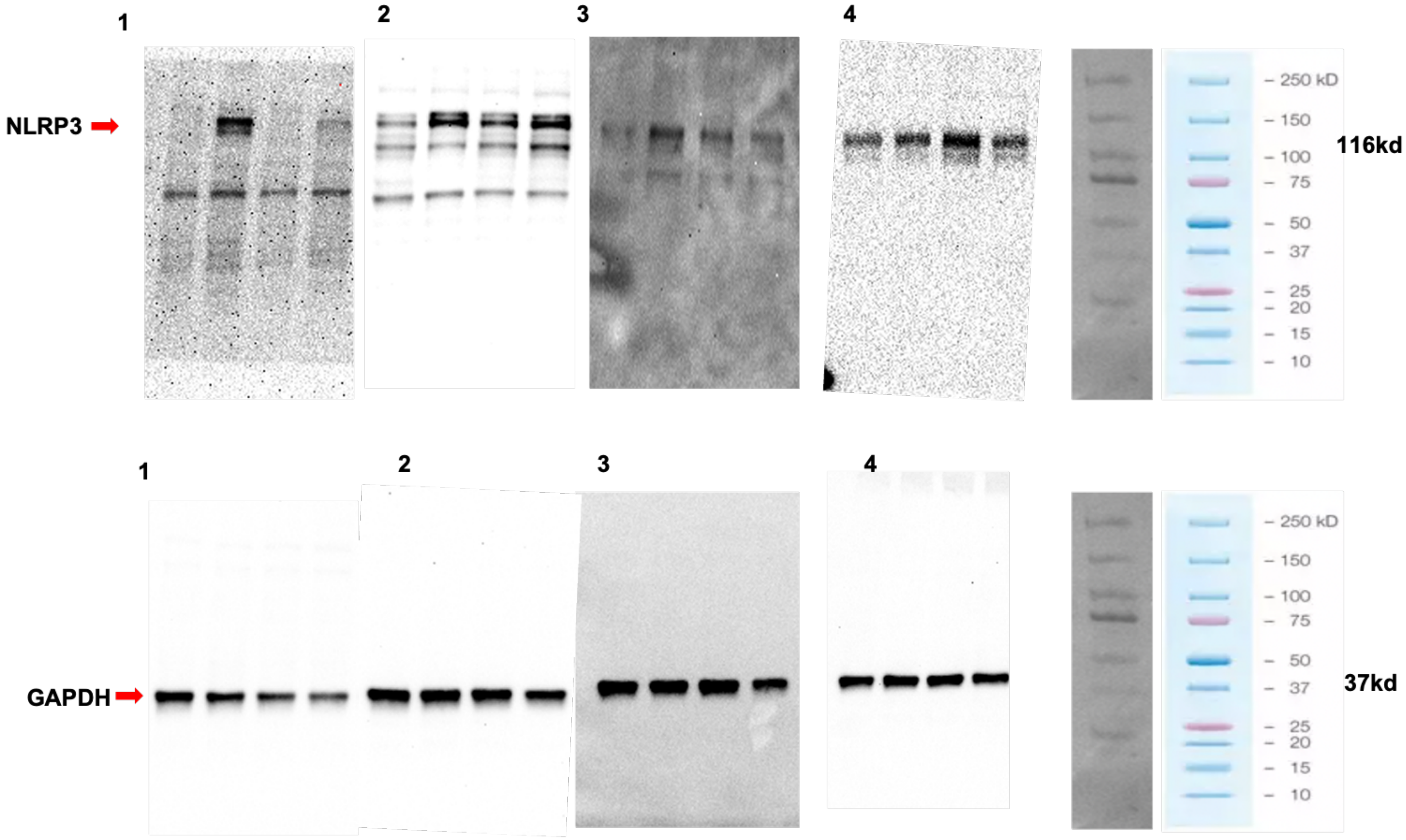

Supplemental figure 5. The uncropped images of the original western blots in figure 4A. Repeated from 4 individual groups.

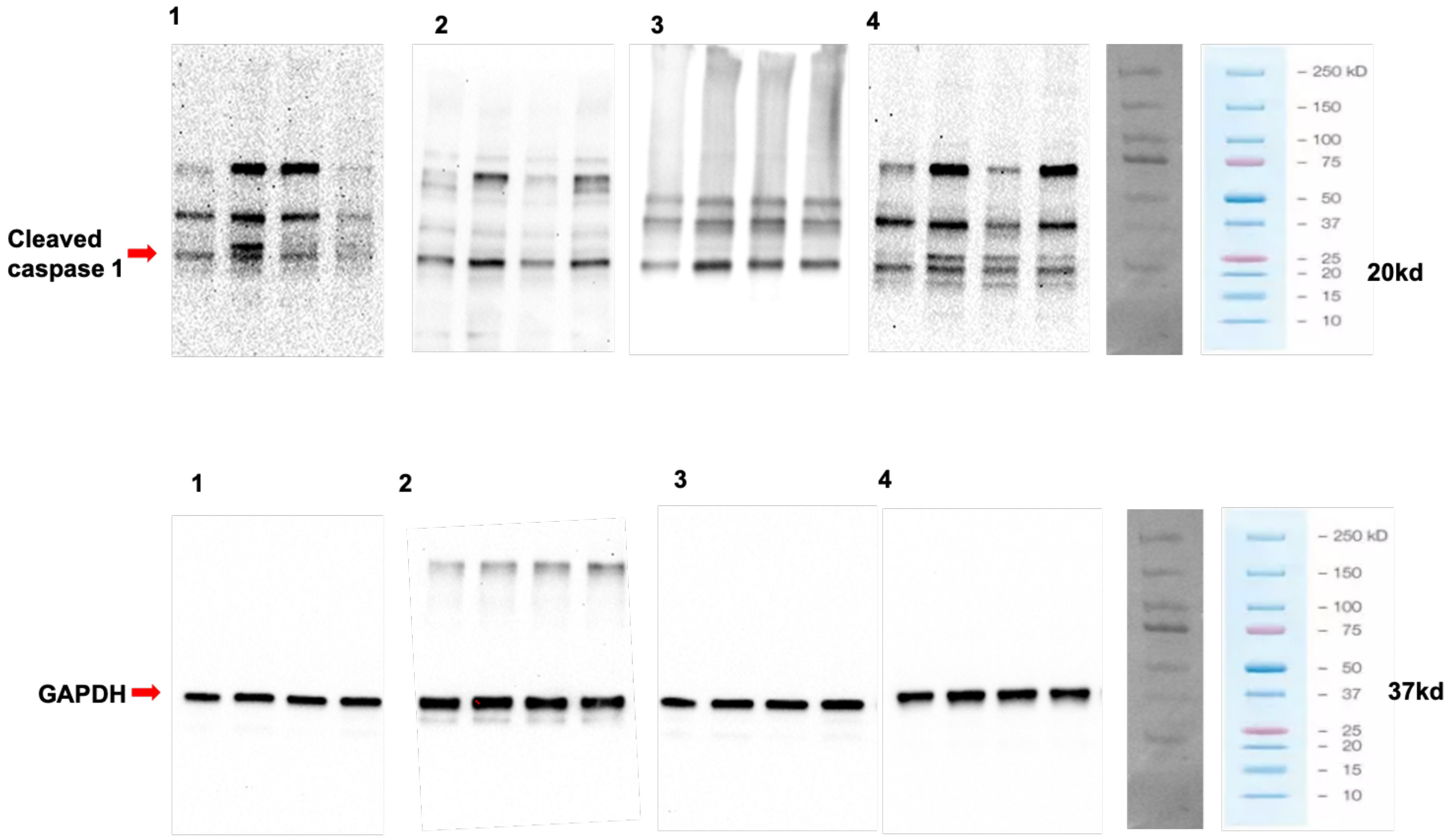

Supplemental figure 6. The uncropped images of the original western blots in figure 4B. Repeated from 4 individual groups.

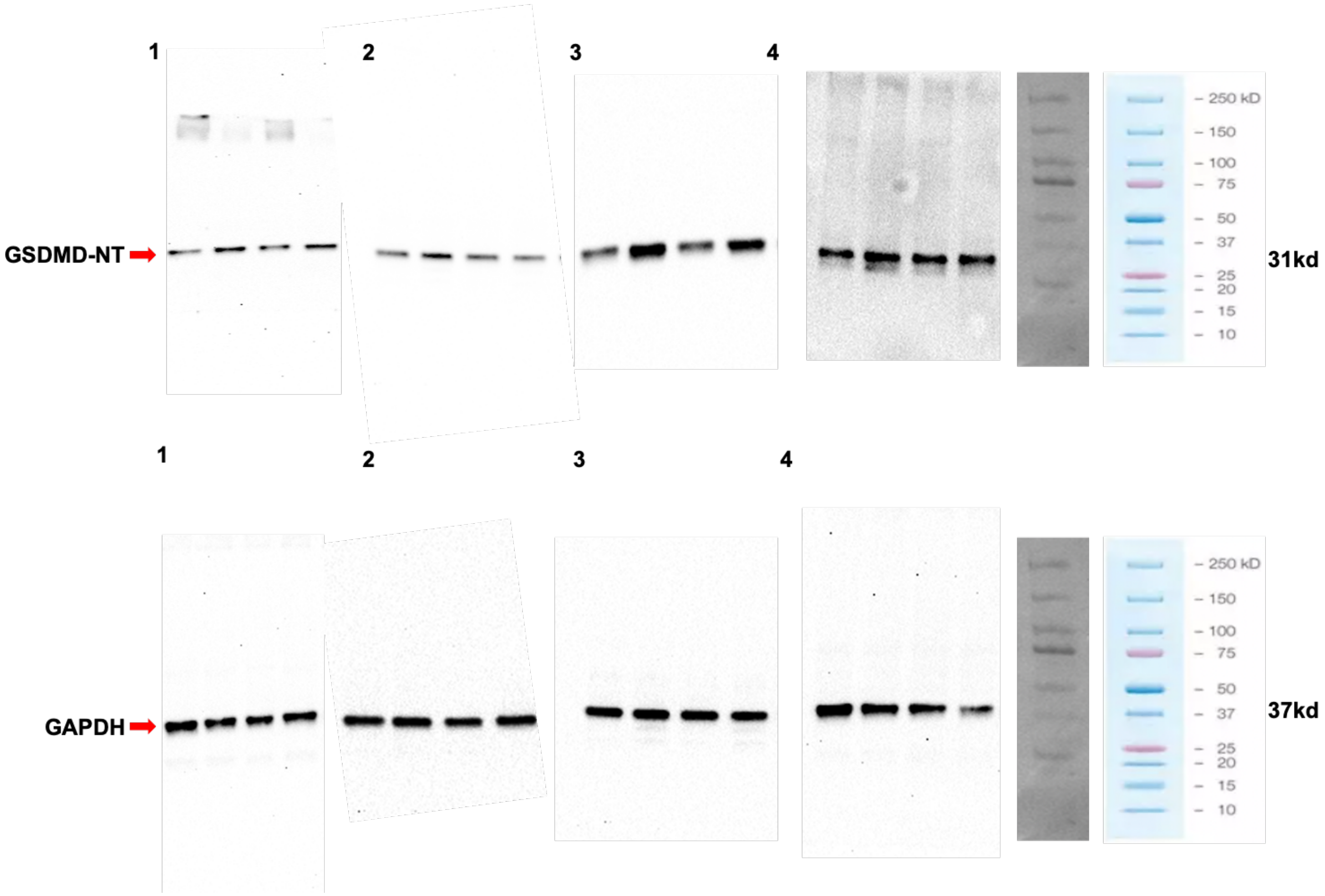

Supplemental figure 7. The uncropped images of the original western blots in figure 4C. Repeated from 4 individual groups.

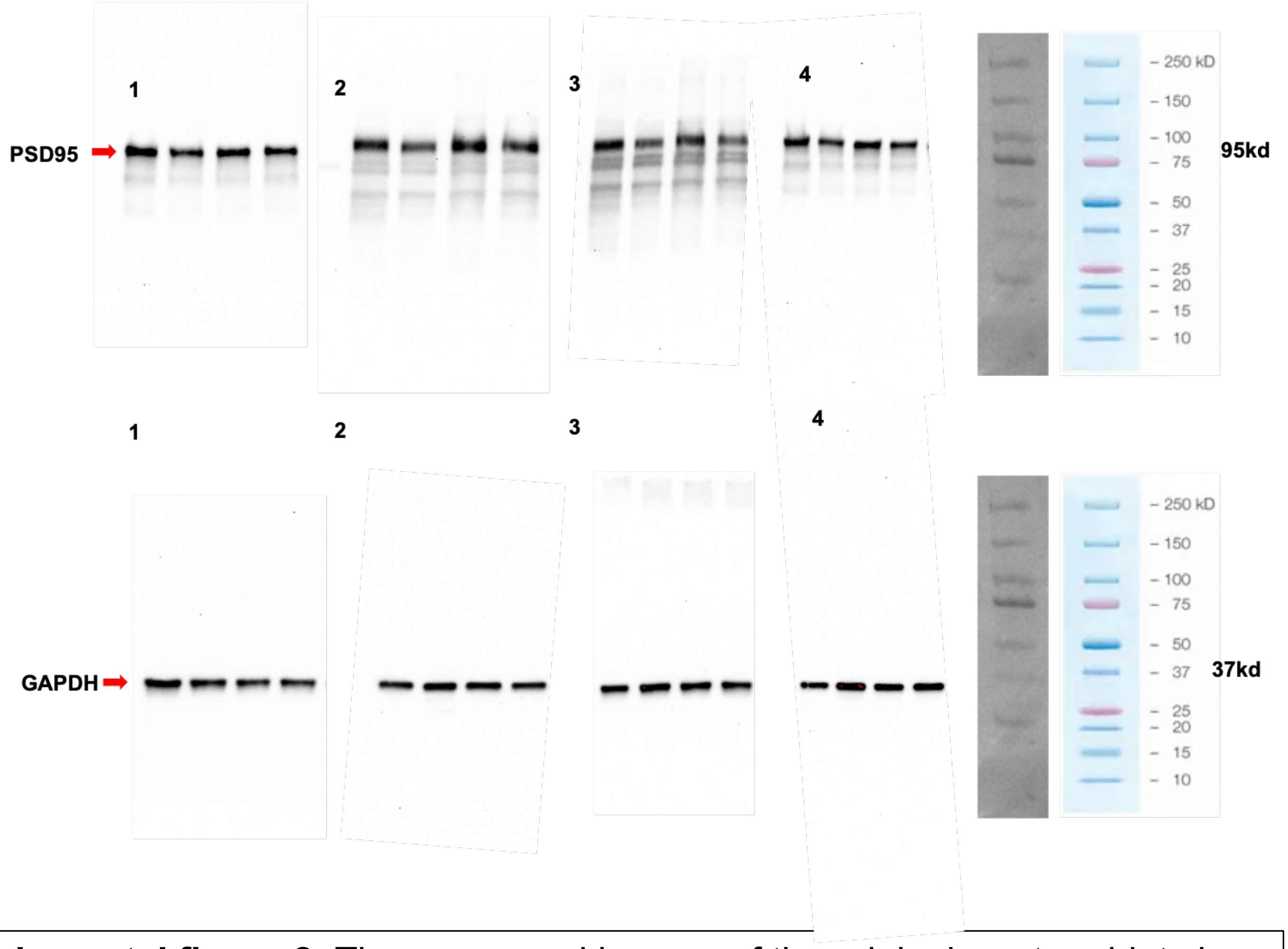

Supplemental figure 8. The uncropped images of the original western blots in figure 5A. Repeated from 4 individual groups.

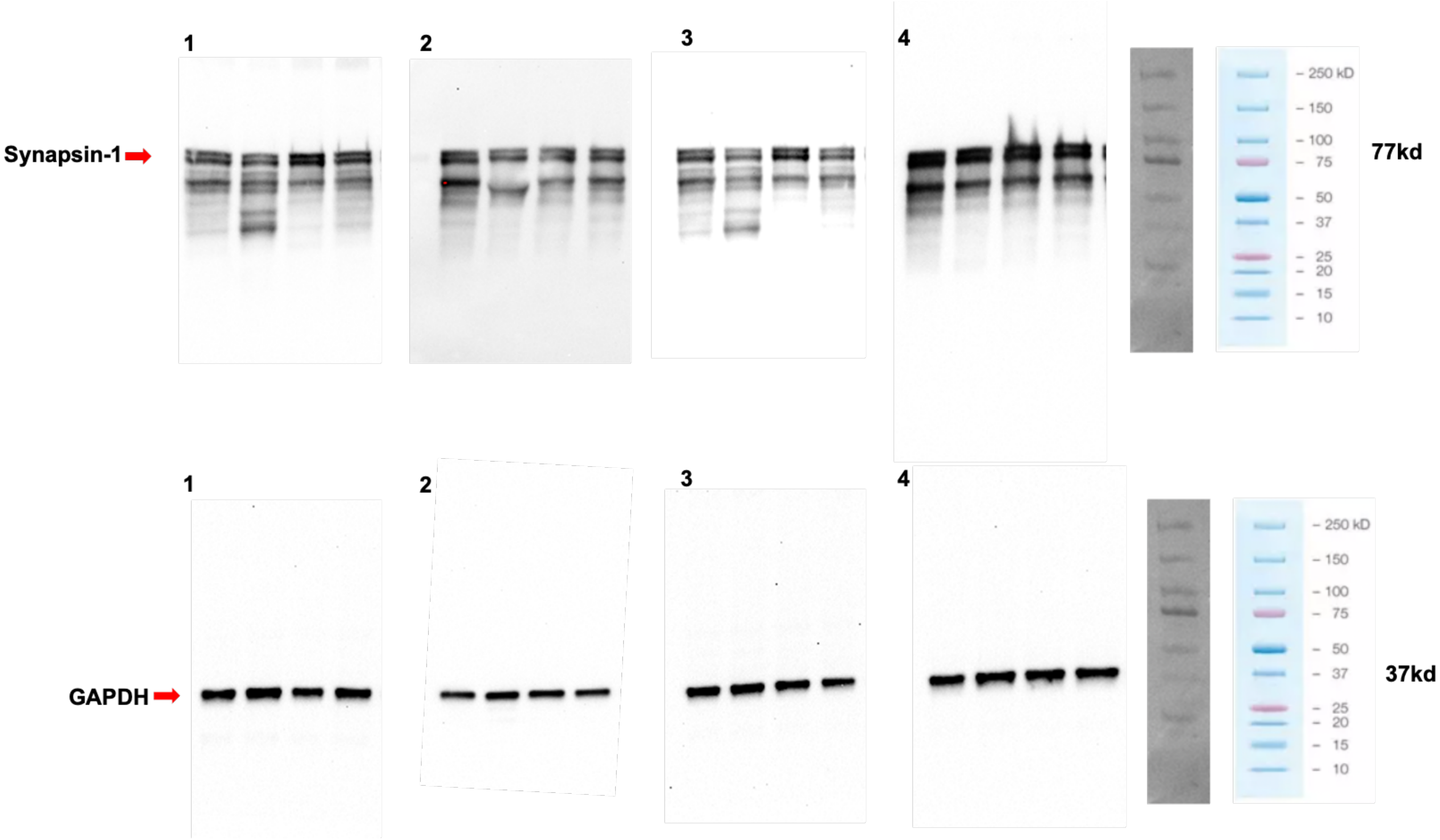

Supplemental figure 9. The uncropped images of the original western blots in figure 5B. Repeated from 4 individual groups.

Table 1	Cat. and Manufacture	Dilution
Primary antibody		
Anti- NLRP3	68102-1-Ig; Proteintech	1:2000
Anti-Human procaspase-1/ cleaved caspase-1 p20	#2225S, Cell signaling Technology,USA	1:1000
Anti- Cleaved GSDMD	#36425S; Cell signaling Technology, USA	1:1000
Anti-IL-1β	31202S, Cell signaling Technology, USA	1:1000
Anti-IL-18	10663-1-AP; Proteintech	1:3000
Anti-PSD95	#2507; Cell signaling Technology, USA	1:1000
Anti-Synapsin-1	#5297; Cell signaling Technology, USA	1:1000
GAPDH	MA5–15738, Thermo Fisher Scientific	1:10,000
Secondary antibody		
Anti-Mouse IgG1; HRP -linked	# PA1-74421, Thermo Fisher Scientific	1:10,000
Anti-rabbit IgG, HRP-linked	#7074; Cell signaling Technology, USA	1:1000
